# An aggregating *U*-Test for a genetic association study of quantitative traits

**DOI:** 10.1186/1753-6561-5-S9-S23

**Published:** 2011-11-29

**Authors:** Ming Li, Wenjiang Fu, Qing Lu

**Affiliations:** 1 1Department of Epidemiology, Michigan State University, B601 West Fee Hall, East Lansing, MI 48824, USA

## Abstract

We propose a novel aggregating *U*-test for gene-based association analysis. The method considers both rare and common variants. It adaptively searches for potential disease-susceptibility rare variants and collapses them into a single “supervariant.” A forward *U*-test is then used to assess the joint association of the supervariant and other common variants with quantitative traits. Using 200 simulated replicates from the Genetic Analysis Workshop 17 mini-exome data, we compare the performance of the proposed method with that of a commonly used approach, QuTie. We find that our method has an equivalent or greater power than QuTie to detect nine genes that influence the quantitative trait Q1. This new approach provides a powerful tool for detecting both common and rare variants associated with quantitative traits.

## Background

In the past decade, extensive genome-wide association studies (GWAS) have been conducted to understand the genetic etiology of complex diseases. However, the common variants identified so far explain only a small fraction of the variations of complex diseases [1]. It now seems clear that the genetic etiology of complex diseases is highly heterogeneous. Some genetic mutations, although individually rare, may impose a high risk for the development of diseases [2]. These rare variants have been the recipients of growing attention by investigators. With the fast development of biotechnology, it is now feasible to genotype rare sequence variations in the general population with unprecedented speed [3]. Meanwhile, statistical methods are greatly needed to detect the association between these genetic variants and common complex diseases.

The most commonly used approach for detecting the association between rare variants and disease outcome is to collapse multiple rare variants into a single “supervariant,” which is tested further as a common variant. Based on this idea, Li and Leal developed a combined multivariate and collapsing (CMC) method for rare variant analysis [4]. This method was further extended for quantitative traits and was implemented in the software package QuTie [5]. In the past two years, a number of collapsing methods that use various strategies have been proposed, and they provide alternatives to the CMC method [6]. Compared to the multivariate analysis of multiple rare variants, these collapsing methods could reduce the degrees of freedom by creating a single supervariant composed of multiple individual rare variants, thus improving the testing power. In addition, testing on a single supervariant could reduce the burden of multiple testing. However, the existing methods also have a few limitations that may affect their performance. Collapsing all the rare variants in the same gene or genomic region, although biologically meaningful, can also introduce nonfunctional variants into the supervariant, which may diminish the signal that the functional variants carry. Intuitively, this limitation can be addressed by collapsing only a subset of the disease-susceptibility rare variants. In what follows, we refer to the collapsing process using trait information as aggregation.

In this paper, we propose an aggregating *U*-test to examine the association between the quantitative traits and multiple genetic variants, including both rare and common variants. First, the method adaptively collapses the disease-susceptibility rare variants into a supervariant; it then searches the supervariant and the remaining common variants for the best multi-SNP (single-nucleotide polymorphism) combination, using a forward selection. We have applied our method to the Genetic Analysis Workshop 17 (GAW17) mini-exome data and compared its performance with QuTie.

## Methods

We have recently developed a forward *U*-test to detect gene-gene interactions using general multisample *U* statistics [7]. Here, we first describe the definition of *U* statistics and then explain the aggregation of rare variants and the forward selection of multi-SNP combinations using *U* statistics.

Suppose that we have a study population of *N* subjects. Let *Y_i_* denote the observed value of the quantitative trait for the *i*th individual (*i* = 1, 2, …, *N*); and let *X_i_* = *X_i1_*, *X_i2_*, … , *X_iK_*, denote the genotypes of *K* SNPs for the *i*th individual, each taking its value from one of the three possible genotypes, *X_ij_* ∈ {*AA*, *Aa*, *aa*}, *j* = 1, 2, …, *K*. Without loss of generality, we assume that *a* is the minor allele, and the first *r* SNPs (*X_i1_*, *X_i2_*, …, *X_ir_*) are rare variants.

### *U* statistics

Suppose that we have *L* multi-SNP genotypes formed by *k* SNPs of interest, denoted as *G*_1_, *G*_2_, …, *G_L_*. A multi-SNP genotype *G_l_* is defined here as a vector of the *k* genotypes that an individual carries (e.g., *g*_1_, *g*_2_, …, *g_k_*). The *k* SNPs and *L* multi-SNP genotypes are selected sequentially out of a total of *K* genotyped SNPs (see the “Forward *U*-test” section for details). Let:(1)

be the set of subjects carrying multi-SNP genotype *G_l_*, and let *m_l_* = |*S_l_*| be the number of subjects in *S_l_*. We measure the trait difference between two sets of subjects *S_l_* and *S_l′_* as:(2)

where the kernel function is chosen as:(3)

*U_l,l′_*, is the summation of all possible pairwise trait comparisons for any two subjects from *S_l_* and *S_l′_*. In the presence of an association, we would expect individuals carrying different multi-SNP genotypes to have different trait values (e.g., those carrying a high-risk multi-SNP genotype would have higher trait values than those carrying a low-risk multi-SNP genotype). Based on *U_l,l′_*, we can form the global *U* statistic. We assume that the expected quantitative trait value of the *L* multi-SNP genotypes decreases with *l* (i.e., ). Practically, we sort the multi-SNP genotypes according to their average trait values (i.e., ). We define the global *U* statistic for *L* sets of subjects with different multi-SNP genotypes as:(4)

where:(5)

Here, the weight parameter *ω_l,l′_* is chosen to account for the number of subjects in each genotype group. This global *U* statistic measures the overall trait differences among individuals from a total number of *L* multi-SNP genotype groups. It is equivalent to Eq. (2) when *L* = 2.

### Aggregation of the rare variants

When dealing with a large number of rare variants, it is likely that a significant proportion of the rare variants will not be associated with a disease; thus collapsing on a selected subset of rare variants will be necessary. Each rare variant can form two single-SNP genotypes, {*g*_1_ = *Aa* | *aa*, *g*_2_ = *AA*}, for which a *U* statistic can be calculated by using Eq. (2). We rank the *U* statistics in decreasing order as *U*_(1)_, *U*_(2)_, …, *U*_(_*_r_*_)_. Assume that *V*_(1)_, *V*_(2)_, …, *V*_(_*_r_*_)_ are the corresponding rare variants in a candidate gene and that *X_i_*_(1)_, *X_i_*_(2)_, …, *X_i_*_(_*_r_*_)_ are their observed genotypes for individual *i*. We start from variant *V*_(1)_, and define a supervariant as:(6)

At each step of the aggregation process, we add a rare variant with the largest *U* statistic to the supervariant. Accordingly, we redefine the supervariant as:(7)

The supervariant in Eq. (7) always forms two different genotypes, {*R_ij_* = 1, *R_ij_* = 0}, for which a *U* statistic can be calculated using Eq. (2). The collapsing procedure stops at step *t*, where the *U* statistics start to decrease (i.e., ).

### Forward *U*-test

A forward *U*-test is used to evaluate the supervariant and other common variants for their joint association with the trait [7]. We start the process by treating all individuals as a single group. In the first step, each common SNP *j* can form two single-SNP genotypes, , in three possible ways, denoted , , and . As a special case, the supervariant can form only two single-SNP genotypes, . This leads to a total of 3(*K* − *r*) + 1 possible grouping strategies, which can be represented by , where  denotes the *l*th multi-SNP genotype at step *s*. A *U* statistic can be calculated for two sets of subjects grouped by . The SNP with the largest *U* statistic value is selected, and the corresponding grouping is recorded.

In the second step, based on the first selected SNP, a second SNP *j*′ is chosen to form four two-SNP genotypes, denoted by {, , , }. We calculate the global *U* statistics for each of these grouping strategies using Eq. (4) and choose the one with the largest *U* statistics. It should be noted that, if the same SNP from step 1 is chosen in step 2, then only three single-SNP genotypes will be formed, denoted by {, , }. As the algorithm moves forward, the *U* statistic is expected to increase until groups cannot be split further. This results in a series of models with different numbers of groups. The best model, with the appropriate number of groups, can be determined by using a 10-fold cross-validation procedure. The *U* statistic of the best model is calculated using the whole data set, and the significance of its association can be obtained using permutation. For each permutation replicate, the same procedure (including the aggregation process and model selection) is applied to calculate the *U* statistics. An empirical *p*-value, which accounts for inflated type I error resulting from model selection, can be calculated by using a large number of permutations.

## Results

We applied the proposed method to analyze the quantitative trait Q1 in the GAW17 mini-exome data. Thirty-nine SNPs, located in nine genes, were associated with trait Q1. The minor alleles of these SNPs were associated with the higher mean of Q1, and their frequencies ranged from 0.07% to 16.5%. We first adjusted the trait by age, using a linear regression model. The residual scores were then used for our association studies. Based on 200 replicates, we conducted a gene-based association study for each of the nine causal genes. For each gene, the traits were permutated 1,000 times, to generate the empirical null distribution of the *U* statistics. We then evaluated the power of our method by counting the number of replicates whose *U* statistics exceeded the 95th percentile of the null distribution. A similar analysis was also conducted using QuTie, version 0.2. The threshold for rare variants was chosen as a minor allele frequency (MAF) less than 0.01. The performance of the two methods varied according to the number of SNPs within the genes, the number of causal SNPs, and their effect sizes. We divided the nine genes into five groups accordingly (Table 1).

**Table 1:** Power comparison of the aggregating *U*-Test and QuTie

Group	Gene	Number of causal SNPs/Total number of SNPs in gene		Power (QuTie)	Power (aggregated *U*)	Type I error (aggregated *U*)
					
		Rare variants	Common variants			
1^a^	*FLT1*	11/35		0.86	1.000	0.036
					
		8/25	3/10			

2^b^	*VEGFC*	1/1		0.745	0.785	0.054
					
		1/1	0/0			

3^c^	*ELAVL4*	2/10		0.025	0.585	0.050
					
		2/7	0/3			
	
	*FLT4*	2/10		0.58	0.715	0.060
					
		2/8	0/2			
	
	*HIF1A*	4/8		0.215	0.915	0.048
					
		3/7	1/1			
	
	*VEGFA*	1/6		0	0.265	0.060
					
		1/5	0/1			

4^d^	*KDR*	10/16		0.99	0.840	0.038
					
		8/14	2/2			

5^e^	*HIF3A*	3/21		0.055	0.05	0.040
					
		3/15	0/6			
	
	*ARNT*	5/18		0.05	0.07	0.038
					
		4/15	1/3			

^a^ Common SNPs within a gene are causal with large effect.

^b^ All rare SNPs within a gene are causal with large effect.

^c^ A small proportion of rare variants are causal.

^d^ A majority of rare variants are causal.

^e^ A small proportion of rare variants are causal, each carrying a small effect.

We found that both methods had a high power to detect the association for genes in group 1 and group 2. As a special case, gene *VEGFC* had only one rare variant, and therefore no selection was necessary. Both methods were able to detect this SNP because of its large effect size. The aggregating *U*-test showed a significant power improvement over QuTie for genes in group 3. For example, gene *ELAVL4* was composed of seven rare variants and three common variants, among which only two rare variants were causal. The individual effects of these variants, though relatively high (0.769 and 0.304), were mitigated by collapsing them with other SNPs, which led to low power by QuTie. In addition, both methods had high power to detect the association for genes in group 4, whereas QuTie attained higher power than the aggregating *U*-test. Because most of the rare variants in the gene were causal and their effects were relatively large, it would be ideal to collapse all the variants. In such a case, the aggregation would have a smaller advantage because the selection process would introduce additional variations. However, we believe that this is not a common scenario in a real data application. Finally, both methods had low power to detect the association for genes in group 5. For both genes, the selection of rare variants did not show any advantage because of the low effect of each functional rare variant.

## Discussion

Our method has two major advantages: (1) It can substantially improve the testing power when only a small proportion of rare variants under examination are functional; and (2) it collapses only a subset of selected rare variants that are potentially trait related. Therefore it can help to identify disease-susceptibility rare variants, which makes our results easier to interpret and replicate in follow-up studies. The existing methods, such as QuTie, collapse all rare variants within the same genomic region and analyze the rare and common variants without differentiating the functional and nonfunctional variants. As illustrated by our analysis, such methods are subject to low power when a significant proportion of the variants in the genomic region are not trait related. Nevertheless, they could have comparable or even higher power over our method when most (or all) of the variants within a gene are trait related. Moreover, because existing methods do not adopt a model selection algorithm to eliminate the noise loci, they are computationally faster than the proposed method. However, if we make the same assumption as existing methods (i.e., that all the variants are associated with disease), then we could also use the asymptotic result of the proposed method to test for association without model selection and permutation [7]. Under the null hypothesis, asymptotically the global *U* statistic has a mean of zero and follows a normal distribution.

We also note that the aggregation of rare variants is different from the forward selection of multi-SNP genotypes. During the aggregation process, the supervariant always forms two genotype groups: one with all rare alleles and the other without any rare allele. The corresponding *U* statistic first increases by adding the risk rare alleles and then decreases when nonrisk rare alleles are added. On the contrary, the number of genotype groups in the forward selection process keeps increasing as the algorithm moves forward, which results in an increasing global *U* statistic (i.e., the model with the largest number of genotype groups has the highest global *U* statistic). Therefore the cross-validation procedure is necessary for forward selection to avoid overfitting the data. The cross-validation procedure, however, is not practical for the aggregation of rare variants, because their low MAFs may cause the absence of rare alleles in the testing set.

## Conclusions

The proposed aggregating *U*-test provides a powerful tool for genetic association studies with both common and rare variants. Our method could also be useful for identifying disease-susceptibility variants underlying quantitative traits.

## Competing interests

The authors declare that there are no competing interests.

## Authors’ contributions

ML developed the method, carried out the analysis and drafted the manuscript. WF participated in the design and helped to draft the manuscript. QL conceived of the study, developed the method, participated in its design and coordination and helped to draft the manuscript. All authors read and approved the final manuscript.
